# Clinical features of atypical odontalgia; three cases and literature reviews

**DOI:** 10.1186/s13030-017-0106-8

**Published:** 2017-08-03

**Authors:** Miho Takenoshita, Anna Miura, Yukiko Shinohara, Rou Mikuzuki, Shiori Sugawara, Trang Thi Huyen Tu, Kaoru Kawasaki, Takeru Kyuragi, Yojiro Umezaki, Akira Toyofuku

**Affiliations:** 1 Department of Psychosomatic Dentistry, Graduate School of Medical and Dental Sciences, Tokyo Medical and Dental University (TMDU), 1-5-45 Yushima, Bunkyo-ku, Tokyo, 113-8549 Japan; 2 Psychosomatic Dentistry Clinic, Dental Hospital, Tokyo Medical and Dental University (TMDU), 1-5-45 Yushima, Bunkyo-ku, Tokyo, 113-8549 Japan

**Keywords:** Atypical odontalgia, Antidepressants, Aripiprazole, Augmentation, Persistent dento-alveolar pain disorder

## Abstract

**Background:**

Atypical odontalgia (AO) is a disease characterized by continuous pain affecting the teeth or tooth sockets after extraction in the absence of any identifiable cause on clinical or radiographic examination. Antidepressants, such as amitriptyline, are reported to be effective in the treatment of AO; however, their efficacy varies depending on the case. In this article, we report three types of AO and discuss its heterogeneity and management.

**Case presentation:**

In the first case, a 58-year-old woman presented with a heavy, splitting pain in the four maxillary front post-crown teeth, as if they were being pressed from the side. Her symptoms abated with 20 mg of amitriptyline. In the second case, a 39-year-old woman presented with a feeling of heaviness pain on the right side of maxillary and mandibular molar teeth, face, whole palate, and throat. She was unable to function because of her pain. Her symptoms drastically subsided with 3 mg of aripiprazole. In the third case, a 54-year-old woman presented with a tingling sensation on the left mandibular second premolar and first molar, and an uncomfortable feeling on her provisional prosthesis that made it unbearable to keep the caps on. Her symptoms diminished with 2 mg of aripiprazole added to 30 mg of mirtazapine.

**Conclusions:**

AO shows various features and responses to drugs. It is considered not only a purely sensory problem, but also a considerably complex psychological problem, such as rumination about the pain. Investigating the difference in pharmacotherapeutic responses might help to advance the treatment of AO.

## Background

Atypical odontalgia (AO), also termed phantom tooth pain [1] or non-odontogenic tooth pain [2], is a disease characterized by continuous pain affecting the teeth or tooth sockets after extraction in the absence of any identifiable cause on clinical or radiographic examination. AO is a specific problem in the dental field, and it seems to be surprisingly complex. Antidepressants, such as amitriptyline, are reported to be effective in the treatment of AO; however, their efficacy varies depending on the case. We often encounter difficult cases where diagnoses are difficult or medications are ineffective.

In this article, we report three types of AO that responded to amitriptyline monotherapy, low-dose aripiprazole monotherapy, or aripiprazole combined with mirtazapine and discuss the its heterogeneity and management.

## Case presentation

Case 1. A 58-year-old female university teacher who was living with her husband was referred to our clinic after complaining of a heavy, splitting pain in the four maxillary front post-crown teeth, as if they were being pressed from the side. Her medical history was unremarkable except for hypertension and hyperlipidemia. She was taking candesartan cilexetil and alprazolam. She had no psychiatric history and no significant family history.

No particular psychological factors could be identified; however, she was anxious about the unexplained pain for a long time.

Five months before the first visit, she had undergone root canal treatment of the left mandibular first molar at a primary dental clinic, and the pain in the maxillary right and left central incisors and lateral incisor appeared 2 months after dental treatment. Afterwards, she underwent examination with radiography at a dental college hospital, but no abnormalities were found, and a CT scan at another dental clinic also revealed no abnormalities. She found our department on the Internet and was referred to our clinic by her primary care physician.

Although the patient had anxiety, obvious signs of depression were absent. Her Zung Self-Rating Depression Scale (SDS) score was 53; however, she did not have depressive mood, lack of emotion, lack of energy, nor suicidal idea. Therefore, we started treatment with 10 mg of amitriptyline and increased the dose to 20 mg 1 week later. Her symptoms started to improve 3 weeks after her first visit. She said, “The character of my pain changed from a feeling of the tooth being broken to a pressure feeling.” Her symptoms were cured one and a half months after her first visit. She continued taking 20 mg of amitriptyline for 4 months, and then the dose was gradually tapered and finally ceased 8 months after the first visit. She experienced a remission in her symptoms.

Case 2. A 39-year-old housewife who was living with her husband was referred to our clinic after complaining of a feeling of heaviness pain on the right side maxillary and mandibular molar teeth, face, whole palate, and throat. She was unable to do her housework and tended to to lie down because of her pain. Her medical history was unremarkable, except for congenital deafness and irritable bowel syndrome. She was taking pregabalin, gabapentin, tandospirone, and bromazepam. Her psychiatric history revealed a panic disorder and her brother had committed suicide because of depression. She told us that she had strong anxiety for a pain appearance.

Three years before the initial visit, she had a cold and received antibiotic treatment for her throat at an otorhinolaryngology clinic. After the treatment, a strong pain began in her throat and spread to the face, teeth, ears and palate. She underwent an MRI examination but no abnormalities were found, and a repeat MRI examination at the pain clinic of a university hospital revealed no abnormalities. Although carbamazepine had been prescribed at the otorhinolaryngology clinic, it was not effective. Gabapentin and pregabalin had been prescribed by the psychiatric department of the university hospital but they were also not effective. She found our department on the Internet and was referred to our clinic by an otorhinolaryngologist.

Although the patient had anxiety, obvious signs of depression were not observed. She did not want to take any tablets, so we started treatment with 3 mg of aripiprazole liquid. Her symptoms started to improve around 3 weeks after her first visit. 1 month after the first visit, she said, “I can go outside to take the trash out.” Two months after the first visit, she said, “The strong pain has turned into a dull pain. I’m now able to take a train.” Although we reduced the dose of aripiprazole from 3 mg to 1.5 mg, her symptoms continued to improve. She was able to go to the gym without thinking about the pain all day. Eight months after the first visit, the dose of aripiprazole was gradually decreased from 1.5 mg to 0.5 mg. Thirteen months after the first visit, the patient stopped taking her medication but remained pain-free for a long time. Five years after the first visit, we received a letter from her saying that she had had a baby, which had been her long-held wish.

Case 3. A 54-year-old housewife who was living with her husband was referred to our clinic after complaining of a tingling sensation on her teeth when they were touched (left mandibular second premolar and first molar), which had been treated, and an uncomfortable feeling on her provisional prosthesis that made it unbearable to keep the caps on. She had a history of dysautonomia, gastritis, pyloric ulcer, and stomach polyps. She was taking mirtazapine, alprazolam, domperidone, and rebamipide. Twenty days before her visit to our department, she had visited a mental health clinic that was introduced to her by her primary physician. The diagnosis of her condition was unclear. She had no other family history. No particular psychological factors could be identified at onset.

Five months before the initial visit, she had visited a dental clinic after a metal inlay on her left mandibular first molar had detached. An extension bridge treatment was recommended and a bridge was attached to her left mandibular second premolar, first molar, and second molar. After that, an uncomfortable feeling and pain were caused by the bridge. She attended another dental clinic, where she underwent pulpectomy of her left mandibular first molar and insertion of a new bridge; however, there were no changes in her symptoms. She consulted another dental clinic and was referred to our university hospital department for endodontics, and she visited them 3 months afterwards. There were no specific problems in her pulp treatment, so she was referred to the pain clinic of our hospital by the department for endodontics. Her symptoms did not change, so she was referred to our department by the pain clinic. She was also referred to psychiatry by her family physician 2 days before her first visit.

**Fig. 1:**
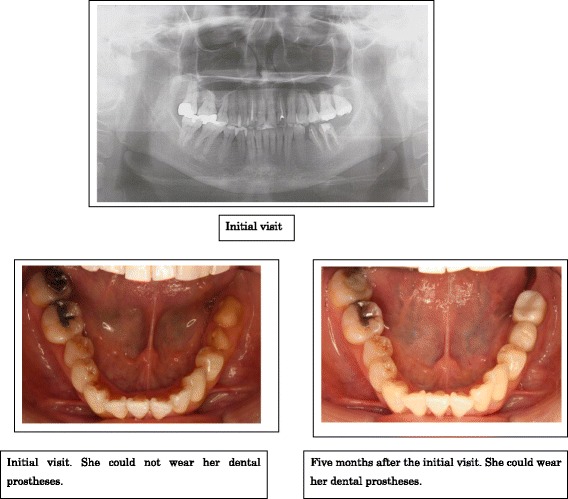
Case 3: panoramic X ray and intraoral findings

Although the patient had anxiety and irritability, obvious signs of depression were not observed. The patient strongly requested a prescription of 7.5 mg of mirtazapine at our hospital, which had been prescribed by the psychiatry department 2 days before her first visit. Thus, we started her pain treatment with 7.5 mg of mirtazapine. After 2 weeks, she said that the pain in her teeth was getting better but the uncomfortable feeling in her provisional teeth remained and she still felt that it was unbearable to keep the caps on. Therefore, we added 1 mg of aripiprazole. Twenty days later, the uncomfortable feeling had slightly improved. Afterwards, we gradually increased the dose of mirtazapine to 30 mg and the dose of aripiprazole to 2 mg, which led to the subsidence of the pain and discomfort. Also, she stated that the time spent thinking about her teeth had decreased. Although the discomfort occasionally reappeared, aripiprazole could be reduced to 1 mg 5 months after her initial visit, and a final prosthesis was attached after the provisional prosthesis (Fig. 1).

## Discussion

As summarized above, it seems that AO has various features. According to the International Association for the Study of Pain, AO is defined as a “severe throbbing pain in the tooth without major pathology” and “persistent (chronic) continuous pain symptom located in the dento-alveolar region and cannot be explained within the context of other diseases or disorders” as a subgroup within persistent idiopathic or atypical facial pain [3]. Recently, AO has been termed persistent dento-alveolar pain disorder (http://www.iasp-pain.org/files/Content/ContentFolders/GlobalYearAgainstPain2/20132014OrofacialPain/FactSheets/Persistent_Dento-Alveolar_Pain_Disorder.pdf) [2, 4]. According to the third edition of the International Headache Classification, it is thought to be a subtype of persistent idiopathic facial pain and is defined as persistent facial and/or oral pain, with varying presentations but recurring daily for more than 2 h per day over more than 3 months, in the absence of a clinical neurological deficit [5]. The definition of AO is being refined and it remains ambiguous [6].

Patients who have AO are predominantly middle-aged women [7–9]. In our cases, the patients were in their 30s to 50s. The onset of symptoms was often after dental treatment but it did not always affect diseased teeth. In case 1, the symptoms appeared in separate teeth. Furthermore, in case 2, the symptoms occurred in a clinical situation that did not involve dental treatment, so the onset of AO seems to vary among individuals. As a matter of fact, post-traumatic peripheral pain neuropathies may occur after dental treatment [10] and 3–6% of patients who had undergone endodontic treatment developed AO [11]. In addition, dental treatment might be a quite stressful event, so it could be a trigger for AO [12].

As for the nature of the pain sensation in AO, our patients complained of a heavy pain, a splitting pain, a tingling sensation, etc., which varied between individuals. The location of the pain also varied between individuals. The nature and location of the pain are similar to those of common dental symptoms such as dental caries, endodontitis, or periodontitis, so it might be difficult for dentists to diagnose AO.

Because of the absence of any organic causes, AO is often regarded as a psychogenic condition, although the relationship between AO and psychologic factors is still unclear [2, 6, 13, 14]. Moreover, the relationship between psychiatric history and AO is uncertain. We previously reported that about 60% of AO patients who were referred to our clinic had been diagnosed with a psychiatric disease [7]. In our cases, AO might have developed regardless of whether or not the patients had a psychiatric history. However, compared with AO patients who had no psychiatric history, AO patients with a psychiatric history might have anticipatory anxiety or rumination about the pain [15] rather than experience of the pain sensation itself. In fact, in case 2, the patient said, “I had a severe pain sensation, but I am also in trouble because I am not able to go out as I am anxious about the pain.” Therefore, AO might not only be simple neuropathic pain but may also have a psychiatric component.

As for the treatment of AO, the effectiveness of antidepressants such as amitriptyline has been reported [9, 13, 14, 16, 17]. Antidepressants activate serotonin and noradrenaline in the nervous system and affect the descending pain inhibitory system of the neurotransmission pathway. However, not all patients respond adequately to antidepressants. In our cases, 20 mg of amitriptyline was effective for pain reduction in case 1. In case 2, 3 mg of aripiprazole was effective for pain reduction. In case 3, although 7.5 mg of mirtazapine was effective for pain reduction, the discomfort remained. Further increase in the dose was unacceptable because of the side effects. Instead, 1 mg of aripiprazole was added, resulting in a remarkable improvement of the discomfort within 3 weeks. As mentioned above, the drug response of AO varies. As seen in case 1, a simple pain sensation might resolve with an antidepressant. As seen in case 2, low-dose aripiprazole was quite effective for the patient’s symptoms. Aripiprazole might be effective not only for the pain sensation but also anticipatory anxiety or rumination about pain. In case 3, a very low dose of aripiprazole in addition to common antidepressants resulted in an improvement. Aripiprazole might be effective for residual uncomfortable feelings. From the findings in our cases, not only serotonin and noradrenaline in the nervous system but also the dopaminergic system might be involved in the pathophysiology of AO.

There is no report of the efficacy of aripiprazole for AO; however, some reports on the treatment of burning mouth syndrome exist [18, 19]. This may be because several neurotransmitter systems, including dopaminergic and serotonergic neural circuits, might be involved with chronic oral pain.

Pain is defined as “an unpleasant sensory and emotional experience associated with actual or potential tissue damage, or described in terms of such damage” [20]. In particular, long-lasting pain can affect various body and brain processes. In other words, it is not the pain in itself, but rather the suffering from persistent pain that evokes various biological and psychological changes, resulting in very complicated and hard-to-treat symptoms. Recently, it has been reported that there is involvement of peripheral and central sensitization of trigeminal pathways in AO [14, 21] as well as a relationship between chronic pain and central sensitization [22, 23]. In addition to these theories, emotional aspects [23] like rumination about the pain might be one of the ways of thinking about AO. There are few treatment-based studies of AO in contrast to psychological studies. Studies of the therapeutic response of AO might be an approach that could reveal its features.

## Conclusion

AO shows various features and responses to drugs. It is considered not only a purely sensory problem, but also a considerably complex psychological problem, such as rumination about the pain. Investigating the difference in pharmacotherapeutic responses might help to advance the treatment of AO. It is hard to diagnose AO precisely and we need an appropriate consensus about AO to prevent overtreatment. Further studies are needed to improve the diagnosis and treatment of AO.
